# Emergence and molecular evolution of carbapenem-resistant hypervirulent ST23 *Klebsiella pneumoniae*: The superbug phenomenon in China

**DOI:** 10.1080/21505594.2025.2545556

**Published:** 2025-08-07

**Authors:** Tao Chen, Xueting Wang, Luying Xiong, Ping Shen, Yonghong Xiao

**Affiliations:** a State Key Laboratory for Diagnosis and Treatment of Infectious Diseases, National Clinical Research Center for Infectious Diseases, China-Singapore Belt and Road Joint Laboratory on Infection Research and Drug Development, National Medical Center for Infectious Diseases, Collaborative Innovation Center for Diagnosis and Treatment of Infectious Diseases, The First Affiliated Hospital, Zhejiang University School of Medicine, Hangzhou, Zhejiang, China; b Jinan Microecological Biomedicine Shandong Laboratory, Jinan, Shandong, China

**Keywords:** *Klebsiella pneumoniae*, hypervirulent, carbapenem-resistant, ST23, China, molecular evolution

## Abstract

This study aimed to investigate the molecular epidemiology of carbapenem-resistant hypervirulent *Klebsiella pneumoniae* (CR-hvKP) ST23 in China. We conducted comprehensive searches across five databases (PubMed, Web of Science, MEDLINE, CNKI, and Wanfang) spanning 1980–2024, identifying 30 eligible studies through rigorous screening. Our synthesis documents 120 ST23 CR-hvKP clinical isolates, including the earliest known case from Zhejiang in 2013 which harbored *bla*_KPC-2_ alongside hallmark virulence loci (*rmpA*, *rmpA2*, *iroN*, *iucA*, and *pagO*). Epidemiological analysis of 119 isolates reveals geographic disparities: Hebei (25.2%) and Jiangxi (22.7%) are hotspots, while carbapenemase distribution exhibits a north–south divide (*bla*_NDM_ predominating in northern China versus *bla*_KPC_ in the south). Phylogenetic analysis of 584 global ST23 genomes suggests independent plasmid-mediated acquisition of carbapenemase genes, with evidence of clonal transmission both among humans and between humans and environmental niches. This review highlights the urgent need for surveillance to track CR-hvKP’s evolving epidemiology, alongside interventions targeting plasmid-driven resistance spread.

## Introduction

Compared to classical *Klebsiella pneumoniae* strains (cKP), strains that have been identified with increasing frequency in recent years and can cause severe infections, such as pyogenic liver abscess, meningitis, brain abscess, and ophthalmitis, in healthy individuals are classified as hypervirulent (hv) [1]. This is attributed to their ability to infect both healthy and immunocompromised individuals, along with a greater propensity to cause invasive infections and subsequent metastatic spread. Notably, sequence type (ST) 23 has been shown to account for the majority of hypervirulent *K. pneumoniae* (hvKP) isolated in mainland China and Taiwan [2–4]. Historically, ST23 hvKP strains were primarily community-acquired and exhibited rare antibiotic resistance. However, recent reports indicate a growing geographic spread, association with healthcare settings, and increasing multidrug resistance [5–7].

In early 2024, the Global Antimicrobial Resistance and Surveillance System on Emerging Antimicrobial Resistance Reporting (GLASS-EAR) called for information following the rising detection of ST23 hvKP isolates carrying carbapenemase genes in multiple countries [8]. The emergence of *K. pneumoniae* isolates exhibiting both hypervirulence and resistance to last-line carbapenems requires the use of alternative antimicrobial treatments, which may be unavailable in many settings. An even greater morbidity and mortality rate can be anticipated if carbapenem-resistant hypervirulent *K. pneumoniae* (CR-hvKP) strains spread within healthcare settings and impact vulnerable patient populations. The World Health Organization (WHO) has now issued a warning following reports from 16 out of 43 responding countries and territories about the presence of ST23 hvKP, with 12 of these also reporting carbapenem resistance [9]. The overall assessment of risk at the global level is considered moderate. However, there may be insufficient information regarding laboratory diagnosis rates, a lack of data on the actual number of infections and hospitalizations, and challenges in surveillance.

This review seeks to consolidate our understanding of this emerging and evolving pathogen in China. We investigate recent developments in ST23 lineage research, emphasizing the molecular features of ST23 hvKP, as well as the epidemiology and molecular evolution of ST23 CR-hvKP within the country.

## Defining hvKP: Current concepts and standards

Currently, clinical microbiology laboratories face challenges in distinguishing hvKP from cKP. Reliable differentiation between these pathotypes is crucial for clinical management, epidemiological surveillance, and research purposes. The gold standard for differentiation involves murine infection models, where hvKP typically demonstrates a median lethal dose (LD_50_) ranging from 10^1^ to 10^7^ colony-forming units (CFU), contrasting with cKP which generally exhibits an LD_50_ exceeding 10^7^ CFU [10]. However, this method remains primarily restricted to research settings due to its impracticality for routine clinical diagnostics. As an alternative approach, the *Galleria mellonella* infection model has been employed to assess virulence potential, though it cannot definitively discriminate between hvKP and cKP [11]. Another proposed method, the string test, detects hypermucoviscosity as a potential marker for hvKP but suffers from insufficient sensitivity and specificity for clinical utility [12]. Certain clinical presentations strongly suggest hvKP infection, including community-acquired liver abscesses, disseminated infections, or infections at atypical sites (e.g. endophthalmitis, necrotizing fasciitis, or central nervous system involvement) in otherwise healthy individuals [13]. Nevertheless, the overlap of hvKP and cKP in common infections (such as pneumonia and urinary tract infections), healthcare-associated cases, and surveillance studies without detailed clinical data underscores the pressing need for a precise diagnostic tool to identify hvKP strains accurately.

Recent advances have enabled broader application of molecular criteria for defining hvKP. Russo and colleagues demonstrated high diagnostic accuracy (> 0.95) for a panel of genotypic markers—*iucA*, *iroB*, *peg-344*, *rmpA*, and *rmpA2*—through comprehensive phenotypic and genotypic analyses of strain cohorts [14]. These biomarkers are typically located on conserved hvKP virulence plasmids, which have been identified as key genetic elements responsible for enhancing the virulence potential of cKP to levels characteristic of hvKP [15,16]. However, the mere presence of these virulence-associated genes does not invariably translate to a hypervirulent phenotype. This distinction prompted Yang et al. to advocate for more cautious use of the term “hypervirulence,” particularly when describing CRKP strains harboring such genetic determinants [17].

## Molecular insights into ST23 hvKP

The first clinical report that highlighted hvKP was a 1986 publication by Liu et al., which described seven cases of invasive *K. pneumoniae* pyogenic liver abscesses in community members in Taiwan [18]. In 2004, Fang et al. found that *K. pneumoniae* strains responsible for hepatic abscesses in patients from Taiwan were more frequently associated with a hypermucoviscous phenotype – characterized by the formation of viscous strings longer than 5 mm, also referred to as a positive string test – compared to non-invasive strains [19]. Since then, hvKP has become highly prevalent in the Asia-Pacific region and among Asian populations, frequently resulting in community-acquired infections, especially in immunocompetent individuals [20]. Its significant pathogenicity contributes to elevated morbidity and mortality rates. hvKP is particularly known for causing liver abscesses, often in conjunction with co-infections of distant organs, such as endogenous endophthalmitis, hematogenous lung abscesses, or brain abscesses. ST23 hvKP is closely linked to the highly serum-resistant K1 capsule and is associated with severe invasive clinical infections [21,22]. A comparative analysis of 97 genomes from clonal group 23 (CG23) strains revealed several sublineages, with CG23-I being the most dominant, representing 81 out of the 97 isolates. The estimated dates for the most recent common ancestors of the entire CG23 population and the CG23-I sublineage were 1878 (1827 – 1915) and 1928 (1908 – 1953), respectively [1].

ST23 hvKP typically contained chromosomal virulence loci *clb* (genotoxin colibactin), *mcc* (microcin E492), *ybt* (yersiniabactin biosynthesis) and the plasmid-associated gene loci [23]. Initial sequencing of the ST23 hvKP strain NTUH-K2044 revealed the presence of a large IncHI1B (pNDM- MAR)/repB-type virulence plasmid, pK2044 (224,152 bp), the loss of which significantly reduced virulence [24,25]. Furthermore, this plasmid harbors virulence determinants that have experimental evidence supporting their role in conferring the hypervirulent phenotype and are strong predictors of an hvKP strain. These include *rmpA* (regulator of mucoid phenotype), *rmpA2*, *iuc* (aerobactin synthesis), and *iro* (salmochelin biosynthesis) [14,26,27]. Strains within the ST23 population generally possess highly similar virulence plasmids resembling pK2044, with variations arising from insertion-deletion events or chromosomal integration. For instance, Ye et al. examined 40 hvKP strains isolated from patients with community-acquired liver abscesses, of which 19 were identified as ST23 hvKP [28]. The plasmid profiles of these 19 strains revealed that 17 contained a single plasmid comparable in size to pK2044 (approximately 220 kb) or with sizes ranging from 140 to 250 kb. The remaining two strains lacked detectable plasmids but carried the *iuc*, *iro*, *rmpA*, and *rmpA2* genes, suggesting that these “plasmid-encoded” virulence genes are integrated into the chromosome. Similarly, a study conducted by Struve et al. showed that all 27 ST23 hvKP strains isolated from liver abscesses or community-acquired pneumonia possessed virulence plasmids containing *iuc*, *iro*, *rmpA*, and *rmpA2*. However, some strains exhibited plasmid variants with specific regions that had undergone deletions [29]. Integrative and conjugative elements (ICEs), a type of mobile genetic element, are commonly found in ST23 hvKP strains. ICEs are primarily known to insert into various asparagine tRNA genes within the *K. pneumoniae* chromosomes [30]. The well-characterized ICE*Kp*1 in the ST23 hvKP strain NTUH-K2044 contains a region homologous to the high-pathogenicity island of Yersinia, which includes the *ybt* genes, as well as a region similar to the virulence plasmid pK2044, containing homologues of the *iro* and *rmpA* genes [31]. Lai et al. described a more widely conserved ICE, ICE*Kp*10, which is typically present in the majority of ST23 hvKP strains [32]. Unlike ICE*Kp*1, ICE*Kp*10 lacks the *rmpA* and *iro* genes but features a 50-kb region that contains *clb* and *mcc* genes. Struve et al. found that all 27 ST23 hvKP strains possessed ICE*Kp*10 homologues, although the *clb* and *mcc* loci were absent in four strains, and the *ybt* locus was deleted in three of those four strains [29]. The acquisition of ICE*Kp*10 and the expansion of ST23 highlight the significant role of this element in the biology of hvKP ST23.

Antimicrobial resistance can be mediated by various mechanisms, each occurring to different extents. Most ST23 hvKP strains demonstrate high sensitivity to the majority of antimicrobial agents, except for intrinsical resistance to ampicillin. However, the acquisition of conjugative plasmids containing antimicrobial resistance determinants – including those for resistance to beta-lactams, aminoglycosides, trimethoprim-sulfamethoxazole, tetracycline, and fluoroquinolones – along with disruptions or mutations in chromosomal genes (e.g. those encoding outer membrane proteins), can confer varying levels of antimicrobial resistance to ST23 hvKP strains [1]. Therefore, the treatment of ST23 hvKP should involve timely implementation of empirical anti-infective therapy considering both local antimicrobial resistance patterns and the specific site of infection, with appropriate antibiotic regimens selected based on the results of antimicrobial susceptibility testing.

## Literature screening and inclusion criteria for ST23 CR-hvKP studies in China

In recent years, plasmids encoding carbapenem resistance or hypervirulence have continually evolved and spread, leading to the emergence of *K. pneumoniae* strains that possess both carbapenem resistance and hypervirulence [33–35]. In 2009, a KPC-2-producing strain of ST23-K1 hvKP was first detected in Poland, with subsequent reports of similar strains emerging globally, particularly in Europe, America, and Asia [36–38]. Given the WHO’s warning about ST23 CR-hvKP, we conducted a systematic epidemiological assessment of ST23 CR-hvKP in China through an exhaustive literature search spanning 1 January 1980 to 25 August 2024 (Figure S1). This systematic review was conducted in accordance with the PRISMA 2020 guidelines. Our search strategy incorporated five major databases: PubMed, Web of Science, MEDLINE, CNKI, and Wanfang, using the following query: (‘‘hypervirulence” [All Fields] OR ‘‘hypervirulent” [All Fields]) AND (‘‘*Klebsiella pneumoniae*” [MeSH Terms] OR ‘‘*Klebsiella pneumoniae*” [All Fields]) AND (‘‘carbapenem resistance” [All Fields] OR ‘‘carbapenem resistant” [All Fields]) AND (‘‘sequence type 23” [All Fields] OR ‘‘ST 23” [All Fields]) AND (“China”). All authors independently performed dual-phase screening: primary screening of titles/abstracts with annual stratification and full-text evaluation of potentially eligible studies. To ensure objectivity, we implemented a consensus-based adjudication process: initial disagreements between two reviewers triggered third-reviewer arbitration and final inclusion required unanimous agreement among all reviewers. Study eligibility was determined using stringent criteria: (1) molecular confirmation of hypervirulence through detection of at least one characteristic virulence marker (*iucA*, *iroB*, *peg-344* [occasionally annotated as *pagO*], *rmpA*, or *rmpA2*); and (2) carbapenem resistance, defined as either: (i) phenotypic resistance (MIC equal to or greater than 4 μg/mL for imipenem/meropenem or meeting or exceeding 2 μg/mL for ertapenem) or (ii) genotypic evidence of carbapenemase production (detection of *bla*_KPC_, *bla*_NDM_, *bla*_OXA-48-like_, *bla*_VIM_, or *bla*_IMP_ genes). After rigorous evaluation, we included 30 studies meeting all criteria (23 English-language, 7 Chinese-language publications). The primary extracted data included year of isolation, geographic origin (province), specimen type, carbapenem resistance determinants, virulence factors, and other associated variables. These organized datasets are presented in Table S1 and will be extensively referenced in subsequent analyses. Among them, six strains from our national surveillance system, the Blood Bacterial Resistant Investigation Collaborative System (BRICS), have not yet had their data published.

## Spatiotemporal distribution and molecular epidemiology of ST23 CR-hvKP in China

**Figure 1.**
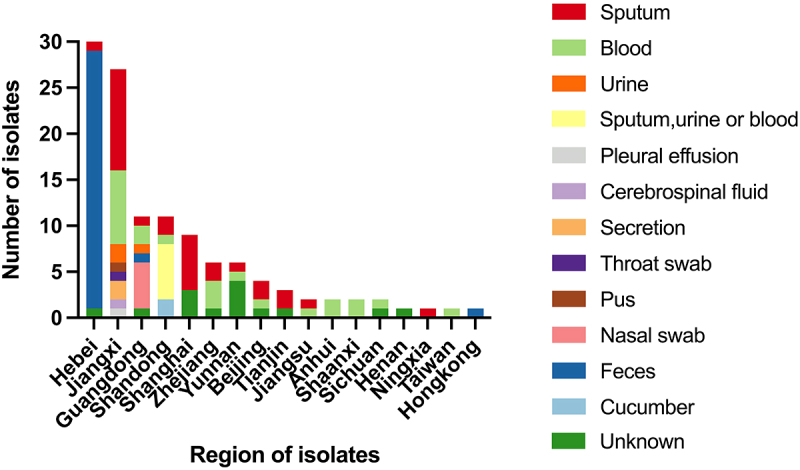
The distribution of specimen sources for CR-hvKP ST23 isolates included in the analysis across different regions (*n* = 119).

A total of 120 carbapenem-resistant *K. pneumoniae* ST23 strains were identified from literature, with 119 strains having available regional data included in this analysis (Figure 1). Among these isolates, the earliest CR-hvKP ST23 isolate was detected in Zhejiang in 2013. This strain carried the *bla*_KPC-2_ gene along with major virulence-associated genes, including *rmpA*, *rmpA2*, *iroN*, *iucA*, and *pagO* [39]. The 119 CR-hvKP ST23 isolates were distributed in Hebei (*n* = 30), Jiangxi (*n* = 27), Shandong (*n* = 11), Guangdong (*n* = 11), Shanghai (*n* = 9), Zhejiang (*n* = 6), Yunnan (*n* = 6), Beijing (*n* = 4), Tianjin (*n* = 3), Shanxi (*n* = 2), Anhui (*n* = 2), Jiangsu (*n* = 2), Sichuan (*n* = 2), Henan (*n* = 1), Ningxia (*n* = 1), Taiwan (*n* = 1), and Hongkong (*n* = 1). Among these, Hebei Province had the highest percentage at 25.2% (30/119), followed by Jiangxi Province at 22.7% (27/119). Shandong Province and Guangdong Province tied for third place, each accounting for 9.2% (11/119) (Figure 1). Among the 119 strains of ST23 CR-hvKP, the sample types were derived from various sources, including sputum, blood, urine, pus, feces and et al. The most common sources were sputum (25.2%, 30/119), feces (25.2%, 30/119) and blood (19.3%, 23/119), respectively. Notably, two strains were isolated from a food sample (cucumber) in Shandong in 2017. These strains exhibited a K1 capsule and encoded KPC-2, showing high resistance to meropenem, with a minimum inhibitory concentration (MIC) value of higher than or equal to 16 mg/L [40]. In the three provinces of Hebei, Jiangxi, and Guangdong, which have the highest sample volumes of ST23 CR-hvKP, the sample types with the highest percentages are feces, sputum, and nasal swabs, respectively. The 119 ST23 CR-hvKP samples mostly originated from community or nosocomial acquired infections, with a small portion originating from healthy human colonization. Among the isolates, 61 with known capsule serotypes belonged to three serotypes: K1, K2, and K5. Similar to the European region, most of the CR-hvKP ST23 isolates in this analysis were identified as capsule serotype K1 (*n* = 58), most of which carry the *rmpA* and *rmpA2* virulence genes, followed by K5 (*n* = 2) and K2 (*n* = 1) [23,41,42]. For a long time, hvKP ST23 (primarily K1/K2) has been recognized as the dominant lineage associated with community-acquired invasive infections, such as liver abscesses with metastatic spread, which can occur in healthy individuals [20]. Two ST23-K5 strains were identified: one was a *bla*_KPC_-positive strain isolated from sputum, and the other was a *bla*_NDM_-positive strain isolated from secretions, both collected in Jiangxi between 2016 and 2018 [43]. The *bla*_KPC_-harboring ST23-K2 strain was isolated from Jiangsu in 2018 [44].

**Figure 2.**
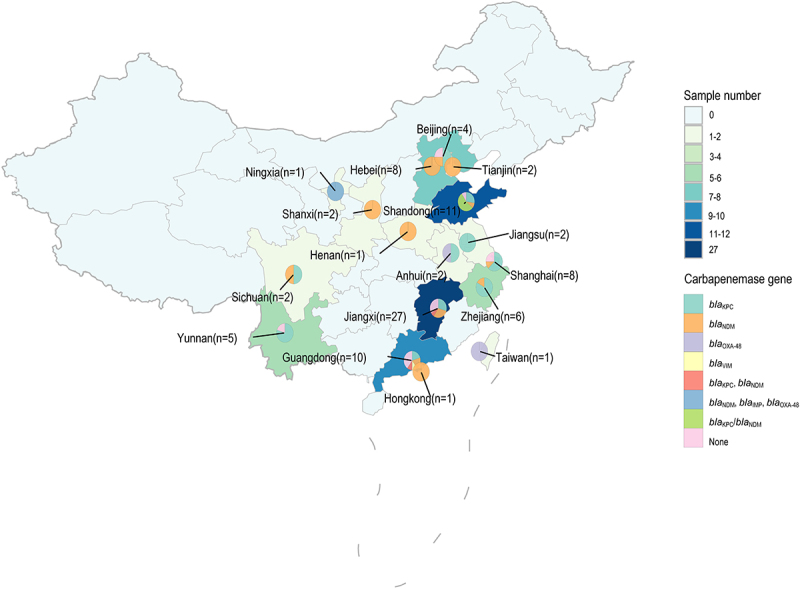
The proportion of carbapenemases among CR-hvKP ST23 isolates in different provinces (*n* = 93).

Among 119 ST23 CR-hvKP isolates analyzed, carbapenemase genes were characterized in 93 strains, revealing *bla*_KPC_ (*n* = 33), *bla*_NDM_ (*n* = 29), *bla*_OXA-48_ (*n* = 2), *bla*_VIM_ (*n* = 1), dual *bla*_KPC_ + *bla*_NDM_ carriers (*n* = 3), and a rare *bla*_NDM_ + *bla*_IMP_ + *bla*_OXA-48_ (*n* = 1) combination, alongside carbapenemase-negative strains (*n* = 18) (Figure 2). Notably, China’s first *bla*_KPC-3_-bearing ST23 CR-hvKP was identified in Zhejiang (March 2021) [45]. Geographic analysis showed non-carbapenemase producers predominated in Guangdong (36.4%, 4/11) and Jiangxi (33.3%, 9/27), while *bla*_NDM_ dominated northern regions (100% in Hebei [8/8], Shanxi [2/2], and Henan [1/1]; 66.7% in Tianjin [2/3]). Conversely, *bla*_KPC_ prevailed in Jiangsu (100%, 2/2), Zhejiang (83.3%, 5/6), Yunnan (80.0%, 4/5), and Shanghai (62.5%, 5/8). Current epidemiological data reveal a distinct geographic divergence in carbapenemase distribution among ST23 CR-hvKP strains across China: *bla*_NDM_-carrying isolates predominate in most northern provinces, whereas *bla*_KPC_-harboring strains are more prevalent in southern regions.

Our analysis identified several clinically significant ST23 CR-hvKP variants, including: (1) a Jiangxi isolate (2016–2018) co-carrying *bla*_KPC-2_ and *bla*_NDM-1_ [43]; (2) *bla*_OXA-48_-positive strains in Anhui and Taiwan [46]; (3) a *bla*_VIM_-carrying strain in Guangdong [47]; and (4) a rare triple-carbapenemase producer (*bla*_NDM_ + *bla*_IMP_ + *bla*_OXA-48_) in Ningxia [48]. These observations suggest that regional carbapenemase prevalence patterns in CRKP may shape the carbapenemase profile of ST23 CR-hvKP clones [39,49]. While China’s ST23 CR-hvKP isolates exhibit diverse carbapenemase genes, *bla*_KPC_ and *bla*_NDM_ remain predominant – a striking contrast to the *bla*_OXA-48_ dominance observed in European Union/European Economic Area (EU/EEA) [38]. However, the observed predominance of *bla*_KPC_ and *bla*_NDM_ is based on epidemiological data derived from published isolates, which likely represent only a subset of the true clinical prevalence. Consequently, comparative analyses of carbapenemase gene distribution among ST23 strains – whether across different regions in China or between China and other geographical locations – remain challenging due to the absence of comprehensive surveillance data and standardized reporting denominators.

**Figure 3.**
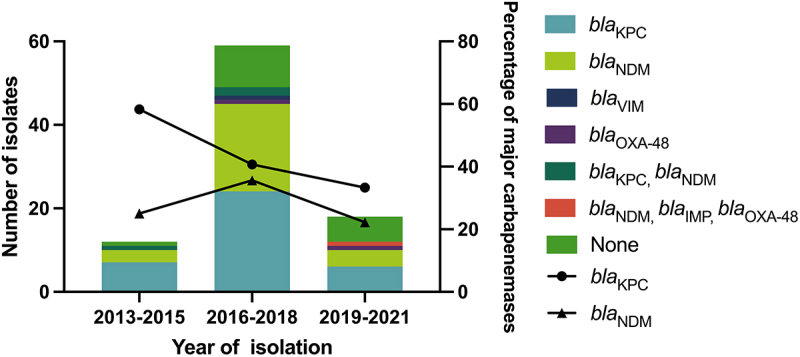
The distribution of carbapenemases in CR-hvKP ST23 isolates by year of isolation (*n* = 89).

Notably, we observed a significant temporal decline in *bla*_KPC_ prevalence among ST23 CR-hvKP isolates between 2013 and 2021. The detection rate of *bla*_KPC_-carrying strains decreased from 58.3% (7/12 isolates) in 2013–2015 to 33.3% (6/18 isolates) in 2019–2021. In contrast, the detection rate of *bla*_NDM_-harboring strains initially increased and then declined (Figure 3). European surveillance data reveal a markedly low prevalence of *bla*_NDM_ among carbapenemase-producing ST23-K1 strains, with only a single case reported to date – an isolate from the Netherlands in 2022 [38]. In contrast, a higher proportion of *bla*_NDM_-harboring strains was found among ST23-K57 strains (47.5%, 19/40) [38]. In China, the detection rate of *bla*_NDM_-harboring ST23-K1 strains was significantly higher than that in Europe, at 31.2% (29/93). The first documented case of *bla*_NDM_-harboring *K. pneumoniae* ST23 dates back to 2014, isolated from a bloodstream infection in Jiangxi Province.

Although *bla*_OXA-48_ is the most commonly detected carbapenemase gene in the hvKP ST23-K1 in the European region, the detection rate of *bla*_OXA-48_ in China is very low, with only two strains of origin of bloodstream infections detected as OXA-48 producers, one isolate in Taiwan in 2018 and the other isolate in Anhui in 2020 [46]. This may be related to the difference in the prevalence of *bla*_OXA-48_-harboring CRKP between Europe and China. In European region, *bla*_OXA-48-like_ was the second most widespread carbapenemase gene found in several STs (266/683, 38.9%), whereas in China, the proportion of *bla*_OXA-48-like_-carrying strains was considerably lower [50]. Moreover, a strain producing multiple carbapenemases (OXA-48, NDM, and IMP) was isolated from a sputum sample of a neonate [48]. For multidrug-resistant and hypervirulent *K. pneumoniae* strains capable of colonization, proactive preventive measures should be taken. This includes reinforcing hand hygiene among clinical staff and enhancing the environmental hygiene in the surrounding area. Additionally, active screening for colonization by resistant strains in both the hospital environment and among patients should be intensified.

Non-carbapenemase-producing CR-hvKP ST23 strain was first detected in 2015, and the proportion has been increasing, accounting for 33.3% (6/18) between 2019 and 2021 [51]. Interestingly, ST23 hvKP can develop carbapenem resistance through a combination of chromosomal mutations and extended-spectrum β-lactamase (ESBL) acquisition, even in the absence of a carbapenemase-encoding plasmid. Specifically, Zhao et al. demonstrated that a resistance plasmid carrying *bla*_CTX-M-71_, in conjunction with a mutated OmpK36 (caused by a single-nucleotide polymorphism, T1076G, resulting in an L359R amino acid substitution), synergistically enhanced carbapenem resistance [52]. This highlights the adaptive capabilities of these bacteria and underscores the importance of understanding alternative resistance mechanisms in combating infections caused by ST23 hvKP. The emergence of non-carbapenemase-producing strains in recent years requires continued monitoring as they do not have major carbapenemase genes and their carbapenem resistance mechanisms are unknown. Rapid identification and differentiation of mechanisms will facilitate optimal treatment and infection control efforts. Increased awareness and vigilance for non-carbapenemase-producing CR-hvKP is needed.

## Genomic insights into the molecular evolution of ST23 CR-hvKP

To elucidate the molecular evolution of ST23 CR-hvKP, we curated a global collection of 584 ST23 *K. pneumoniae* genome assemblies spanning 1980–2023, sourced from 40 geographical regions through the NCBI GenBank database. (see Table S2). The capsular polysaccharide locus (KL) was determined using Kleborate v2.0.147. For core genome analysis, all sequences were aligned to the ST23 reference genome NTUH-K2044 (GenBank accession AP006725.1) using Snippy v4.6.0 (https://github.com/tseemann/snippy). Recombination regions were identified and removed using Gubbins v2.4.1 (https://github.com/nickjcroucher/gubbins), generating both multiple sequence alignments (MSAs) and core genome single nucleotide polymorphisms (cgSNPs). From the resulting 33,204 recombination-filtered cgSNPs, we constructed a maximum-likelihood phylogenetic tree using IQ-TREE2 (https://github.com/iqtree/iqtree2) with a generalized time-reversible (GTR + G) substitution model. Phylogenetic visualization was performed using the Interactive Tree of Life (iTOL) platform (https://itol.embl.de/). Additionally, a minimum spanning tree (MST) was generated in PHYLOViZ 2.0 (https://www.phyloviz.net/) through pairwise cgSNP comparison. The potential transmission clusters were based on the threshold of 25 cgSNPs [53]. Geographic information was available for 575 of these isolates, with the majority originating from China (*n* = 100), followed by Japan (*n* = 76) and Vietnam (*n* = 61). Among the 535 isolates with known origins, the vast majority were isolated from human sources (*n* = 507), while the remaining isolates were primarily obtained from livestock (*n* = 12) and environmental samples (*n* = 16). Of the isolates, 104 encoded one carbapenemase, while 14 strains encoded two carbapenemases (co-producing NDM-1 and OXA-48). Among those encoding a single carbapenemase, KPC-2 was the most frequent (*n* = 56), followed by OXA-48 (*n* = 18) and NDM-1 (*n* = 15).

**Figure 4.**
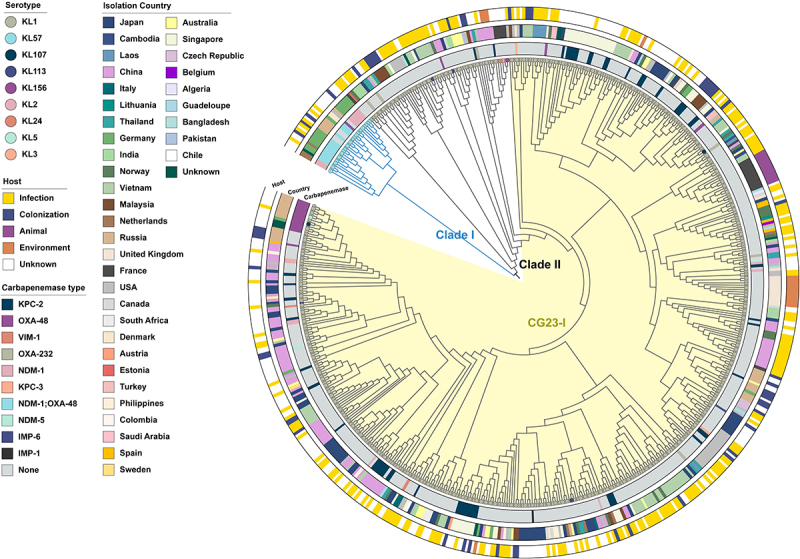
A recombination filtered core genome phylogeny of 584 global ST23 isolates. The colored rings, from the inside out, represent the carbapenemases, the countries of origin, and the host sources of the strains. The CG23-I sublineage is highlighted in the yellow-shaded region of the phylogeny.

**Figure 5.**
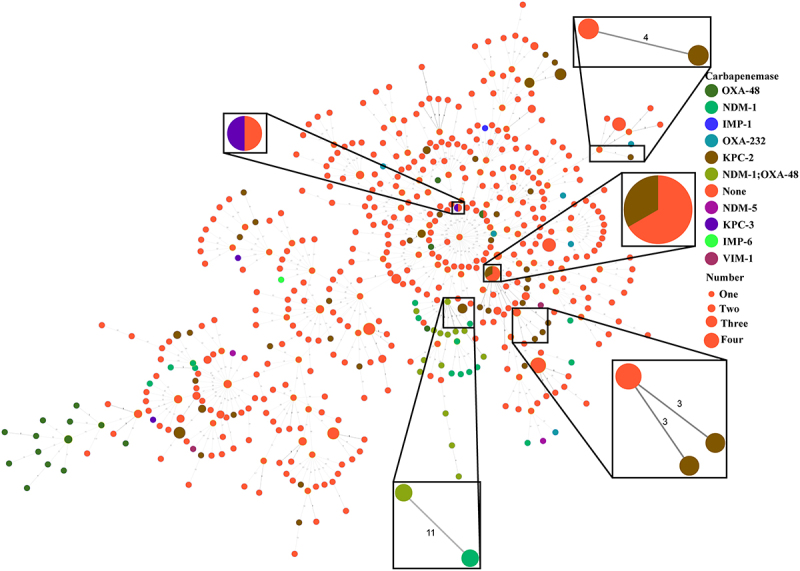
The minimum spanning tree (MST) of 584 global ST23 isolates. Each filled circle represents a different number of ST23 isolates, with the size of the circle proportional to the number of isolates. The number of core genome single nucleotide polymorphisms (cgSNPs) in pairwise comparisons is indicated on the connecting lines.

The phylogenetic analysis revealed two distinct clades (Figure 4). Clade I is composed of ST23-KL57 isolates, which are mainly found in Europe, including Germany, the Netherlands, and Russia. Most of these isolates carry NDM-1/OXA-48 or NDM-1. Clade II, on the other hand, consists mostly of ST23 isolates with the KL1 serotype, interspersed with other serotypes such as KL2, KL3, KL5, KL24, K107, KL113, and KL156. Notably, the previously characterized CG23-I sublineage – typically associated with ICE*Kp*10 and *ybt1*—was nested within Clade II [1]. Previous studies have established that both the KL and lipopolysaccharide synthesis loci represent recognized recombination hotspots in *K. pneumoniae* genomes, frequently resulting in serotype-switching events among phylogenetically closely related subclones [54–56]. Phylogenetic analyses reveal genomic clustering patterns of ST23 Clade II consistent with putative serotype switching events, likely mediated by recombination within the KL locus. Moreover, some closely related isolates from the same country cluster together in the phylogenetic tree, indicating potential spread within national borders may occur. Intriguingly, certain clusters in the phylogenetic tree can be divided into two groups: one harboring carbapenemase genes and the other lacking them. In addition, MST indicates that five groups of strains presenting similar situation (one with carbapenemase and the other not) displays pairwise cgSNPs <25 (Figure 5). These findings further support the hypothesis that the emergence of ST23 CR-hvKP is driven by the horizontal transfer of carbapenem resistance genes to ST23 hvKP strains. The wide distribution of isolates with and without carbapenemase genes throughout the MST indicates that the acquisition of carbapenemase genes has likely occurred independently on multiple occasions, possibly through the acquisition of resistance plasmids. Additionally, some isolates from infected patients cluster with those from environmental and human carriage sources (cgSNPs <25), suggesting the potential for transmission of ST23 hvKP between the environment and humans, and highlighting the significant role of human carriage in the transmission dynamics of ST23 hvKP (Figure S2). Enhanced surveillance is essential to better understand the factors driving intestinal carriage and the transmission dynamics of hvKP, thereby informing more effective preventive measures.

## Diversity of carbapenemase plasmids in ST23 CR-hvKP from China

Various carbapenemase-encoding plasmids play a crucial role in the molecular evolution of ST23 CR-hvKP. Specifically, IncFII(pHN7A8)/IncR, IncFIB(AP001918), repB(R1701), and IncP1 plasmids have been identified as carriers of *bla*_KPC_ genes, while IncX3 plasmids are primarily associated with *bla*_NDM_ genes. The IncFII(pHN7A8)/IncR plasmid is the most common type carrying *bla*_KPC-2_ in ST11 CRKP in China [57]. Notably, a Chinese ST23 CR-hvKP isolate, C4599 (Accession: SAMN37347871), harbors a 128,837-bp *bla*_KPC-2_-carrying IncFII(pHN7A8)/IncR plasmid, which shows 100% identity and 95% coverage to the IncFII(pHN7A8)/IncR plasmid in an ST11-KL47 CRKP isolate, WCHKP8F4 (SAMN06671960), from China. This underscores the significant role of these IncFII(pHN7A8)/IncR plasmids in the development of carbapenem resistance in ST23 hvKP. Similarly, another ST23 CR-hvKP isolate, C1356 (SAMN37347794), from Beijing, carries a 123,239-bp IncFIB(AP001918) plasmid with *bla*_KPC-2_, which has the best match (99.96% identity, 86% coverage) to a *bla*_KPC-2_-carrying IncFIB(AP001918) plasmid in an ST15 CRKP isolate, GZKP13 (SAMN29388304), also from China. This hints a likely horizontal transfer of an IncFIB(AP001918) plasmid carrying the *bla*_KPC-2_ gene from ST15 CRKP to ST23 hvKP strains, accompanied by various gains and losses of plasmid regions. Furthermore, a 105,071-bp *bla*_KPC-2_-carrying repB(R1701) plasmid in the ST23 CR-hvKP strain 1088 (SAMN07259332) from Hong Kong displays a high similarity (> 99.96% identity, > 99% coverage) to the repB(R1701) plasmids found in *Serratia marcescens* isolate S1 (MN615880, China), *Klebsiella aerogenes* isolate C91664 (SAMN38396362, China), and ST15 CRKP isolate P1954 (SAMN18746132, China). This emphasizes the capability of repB(R1701) plasmids to spread across different bacterial species and their significant role in the development of carbapenem resistance in ST23 hvKP. Additionally, the ST23 CR-hvKP isolate ZJ27003 carries a 36,708-bp *bla*_KPC-2_-harboring plasmid, classified as IncP1. This plasmid is similar to a *bla*_GES-5_-harboring *Pseudomonas aeruginosa* plasmid (MH053445.1, China), where the *bla*_GES-5_ and its surrounding regions were replaced by a *bla*_KPC-2_-containing translocatable unit derived from Enterobacteriaceae [34]. These findings collectively indicate that the genetic diversity of acquired KPC-harboring resistance plasmids contributes significantly to the development of carbapenem resistance in ST23 hvKP. In contrast, the *bla*_NDM_ gene in ST23 CR-hvKP is predominantly associated with IncX3 plasmids. For example, a 54,034-bp *bla*_NDM-1_-containing IncX3 plasmid in the ST23 CR-hvKP isolate C1928 (SAMN37347822) from Tianjin shows a high similarity (99.99% identity, 100% coverage) to *bla*_NDM-1_-containing IncX3 plasmids in *Klebsiella aerogenes* KAE3SP (SAMN20964956, China), *Citrobacter freundii* C46 (MW269623, China), and ST1 CRKP SCKP020135 (SAMN07609088, China). This suggests that this prevalent *bla*_NDM-1_-containing plasmid can spread across multiple species and plays a significant role in the development of ST23 CR-hvKP.

## Trade-offs between virulence and antibiotic resistance

The emergence of ST23 CR-hvKP can be attributed to the horizontal transfer of plasmids carrying carbapenem resistance genes from CRKP to ST23 hvKP strains [58]. The acquisition of the carbapenem resistance phenotype by hvKP is probably linked to regional epidemic carbapenem resistance mechanisms. Previous study has found that KPC is the most common type of carbapenemases and is predominant among CRKP strains in China (89.6%, 712/794), followed by NDM (4.5%, 36/794) [59]. These strains widely spread across healthcare facilities in China, resulting in hospital-acquired infections. Interestingly, despite the high prevalence of *bla*_KPC_-harboring CRKP strains in China, the detection rate of *bla*_KPC_-harboring strains in CR-hvKP ST23 is significantly lower (35.5%, 33/93). Given that ST11 is the predominant clone of CRKP, this phenomenon may be associated with the differential abilities of ST23 and ST11 strains to acquire *bla*_KPC_-encoding plasmids. It has been observed that capsule expression correlates with a reduction in transformation and conjugation frequencies of *K. pneumoniae in vitro* [60]. Furthermore, the capsule is known for its capacity to mask lipopolysaccharides (LPS), which is considered essential for the attachment of conjugative pili during the early stages of mate-pair formation [61]. This suggests that hypervirulent clones are evolutionarily constrained by a key aspect of their virulence phenotype, the thick capsule, which in turn limits their adaptability to antimicrobial pressures. This characteristic of K1/K2 hvKP strains likely serves as a barrier against the acquisition of carbapenem resistance plasmids, thus contributing to their lower prevalence among these highly virulent types. Another reason may be the differences in sources: a portion of ST23 CR-hvKP strains is community-acquired, whereas classical CRKP strains are almost hospital-acquired. This difference may result in community-acquired ST23 CR-hvKP not acquiring KPC plasmids from the CRKP strains that are widely spread in hospitals.

A genomic analysis of global *K. pneumoniae* strains indicates that CR-hvKP strains typically emerge when classical *K. pneumoniae* strains acquire hypervirulence genes or plasmids, rather than through the introduction of carbapenem resistance genes into hvKP strains [62]. However, the development of ST23 CR-hvKP follows the latter mechanism. Tian et al. reported that the coexistence of virulence plasmids and newly introduced KPC plasmids within the same ST23 clone did not result in significant resistance to carbapenem antibiotics [63]. However, the loss of RfaH, a transcriptional anti-terminator essential for capsule synthesis, can lead to high carbapenem resistance in ST23 CR-hvKP. These results highlight the evolutionary trade-offs between virulence and antibiotic resistance, and suggest that certain non-antibiotic strategies, such as selecting for capsule inactivation, might inadvertently promote the acquisition of antimicrobial resistance genes.

## Conclusions

Currently, we are observing the convergence of drug-resistant and hypervirulent pathotypes within the same strain, posing a significant threat to public health. ST23 hvKP can acquire resistance to last-resort antibiotic, carbapenem, by multiple mechanisms, including acquiring a carbapenem resistance plasmid. Addressing ST23 CR-hvKP requires a holistic strategy that includes clinical management – such as enforcing strict antimicrobial stewardship, exploring the advantages of combination antibiotic therapies, and prioritizing the development of new antimicrobial agents – and infection control measures. These measures should involve comprehensive surveillance systems, consistent and thorough environmental cleaning, and extensive training and education for healthcare personnel. Additionally, we emphasize the urgent need for more prospective, multicenter, and international studies. Such efforts will be crucial in formulating effective infection control strategies to prevent the further spread of CR-hvKP.

## Supplementary Material

Figure S1.jpeg

Figure S2.tif

## Data Availability

The data supporting the findings of this study, including Table S1 and Table S2, are openly available in Figshare at http://doi.org/10.6084/m9.figshare.28334048 [64]. Additionally, a completed PRISMA checklist has been deposited in the same repository. The review was not registered, and a protocol was not prepared.
