# Supplementary figures and images for: Emergence and molecular evolution of carbapenem-resistant hypervirulent ST23 Klebsiella pneumoniae: The superbug phenomenon in China
Source: Virulence. 2025 Aug 7;16(1):2545556. doi: 10.1080/21505594.2025.2545556 (PMC12351723; doi:10.1080/21505594.2025.2545556)

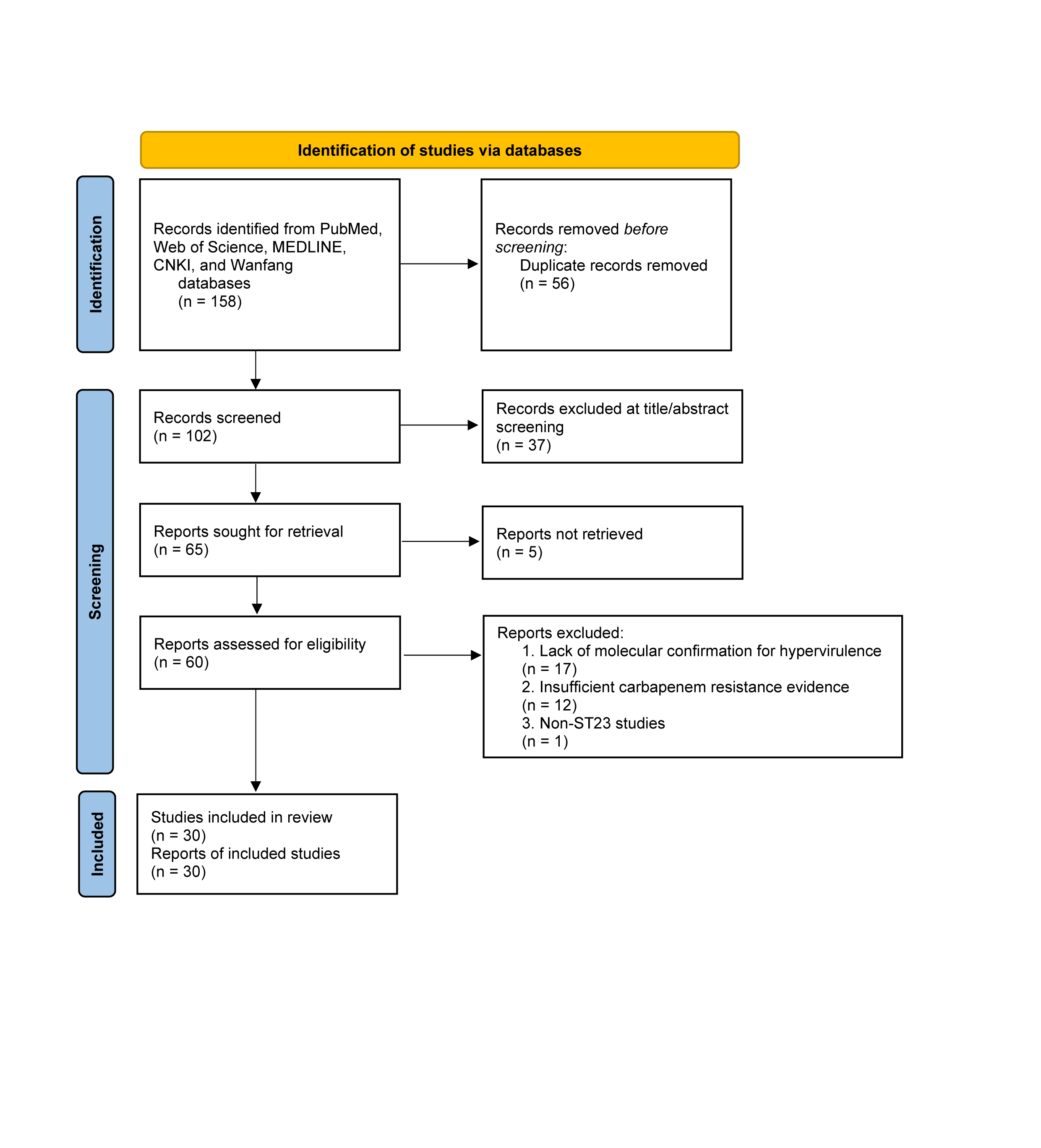

Supplement: Figure S1.jpeg [file KVIR_A_2545556_SM3859.jpeg]

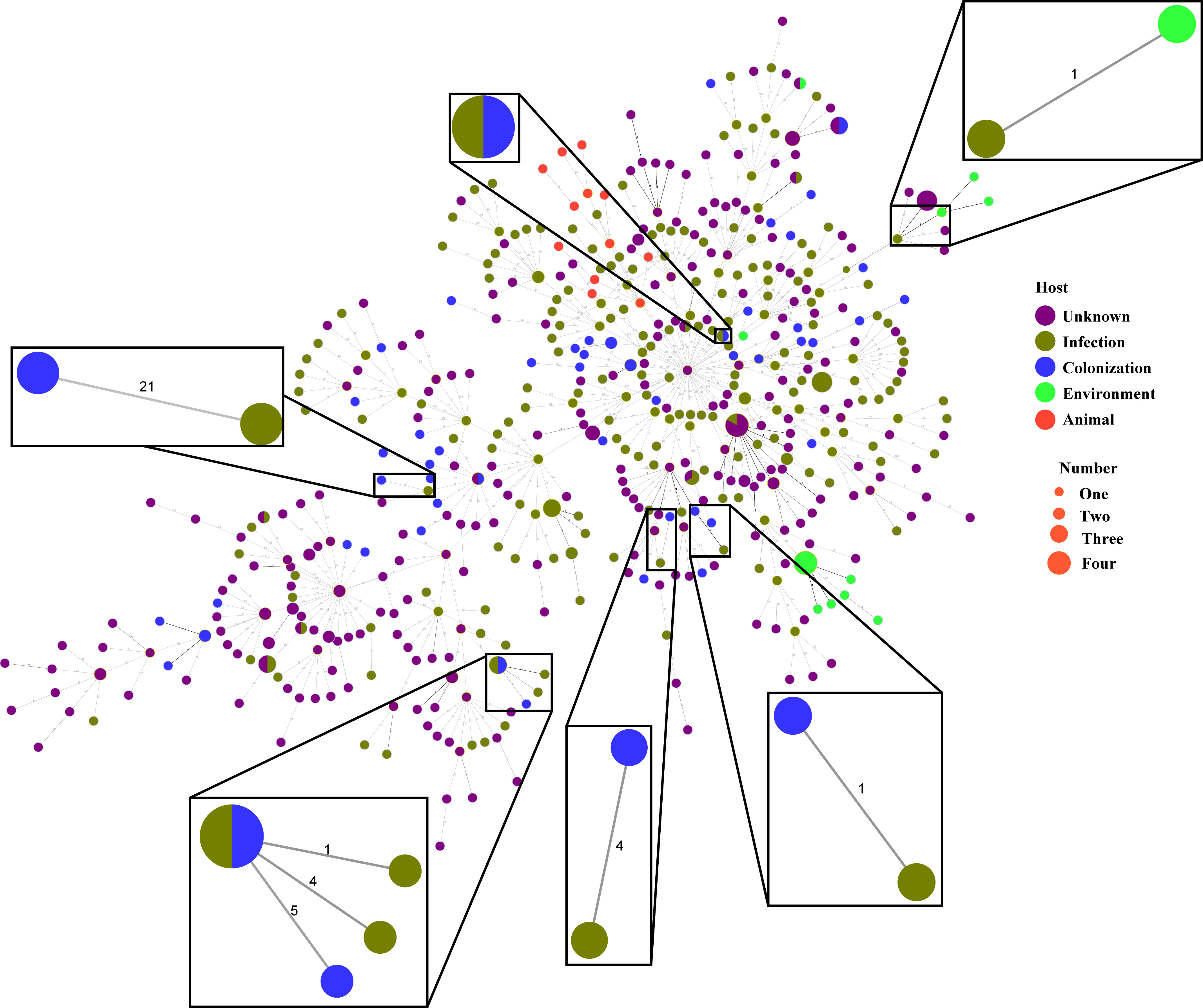

Supplement: Figure S2.tif [file KVIR_A_2545556_SM3858.tif]
